# Newborn resuscitation simulation training and changes in clinical performance and perinatal outcomes: a clinical observational study of 10,481 births

**DOI:** 10.1186/s41077-022-00234-z

**Published:** 2022-11-05

**Authors:** May Sissel Vadla, Robert Moshiro, Paschal Mdoe, Joar Eilevstjønn, Jan Terje Kvaløy, Barikiel Hhando Hhoki, Hege Ersdal

**Affiliations:** 1 Faculty of Health Sciences, University of Stavanger, 4021 Stavanger, Norway; 2 Muhimbili National Hospital, P.O Box 65000, Dar es Salaam, Tanzania; 3 Haydom Lutheran Hospital, Box 9000, Haydom, Mbulu Tanzania; 4 Laerdal Medical, 4002 Stavanger, Norway; 5 Department of Mathematics and Physics, University of Stavanger, 4036 Stavanger, Norway; 6 Department of Research, Stavanger University Hospital, 4011 Stavanger, Norway; 7 Department of Anaesthesia, Stavanger University Hospital, 4011 Stavanger, Norway

**Keywords:** Helping Babies Breathe 2nd Edition, Implementation, In situ simulation-based training, Local champions, Perinatal mortality

## Abstract

**Background:**

Annually, 1.5 million intrapartum-related deaths occur; fresh stillbirths and early newborn deaths. Most of these deaths are preventable with skilled ventilation starting within the first minute of life. Helping Babies Breathe is an educational program shown to improve simulated skills in newborn resuscitation. However, translation into clinical practice remains a challenge. The aim was to describe changes in clinical resuscitation and perinatal outcomes (i.e., fresh stillbirths and 24-h newborn deaths) after introducing a novel simulator (phase 1) and then local champions (phase 2) to facilitate ongoing Helping Babies Breathe skill and scenario simulation training.

**Methods:**

This is a 3-year prospective before/after (2 phases) clinical observational study in Tanzania. Research assistants observed all deliveries from September 2015 through August 2018 and recorded labor/newborn information and perinatal outcomes. A novel simulator with automatic feedback to stimulate self-guided skill training was introduced in September 2016. Local champions were introduced in October 2017 to motivate midwives for weekly training, also team simulations.

**Results:**

The study included 10,481 births. Midwives had practiced self-guided skill training during the last week prior to a real newborn resuscitation in 34% of cases during baseline, 30% in phase 1, and 71% in phase 2. Most real resuscitations were provided by midwives, increasing from 66% in the baseline, to 77% in phase 1, and further to 83% in phase 2. The median time from birth to first ventilation decreased between baseline and phase 2 from 118 (85–165) to 101 (72–150) s, and time pauses during ventilation decreased from 28 to 16%. Ventilations initiated within the first minute did not change significantly (13–16%). The proportion of high-risk deliveries increased during the study period, while perinatal mortality remained unchanged.

**Conclusions:**

This study reports a gradual improvement in real newborn resuscitation skills after introducing a novel simulator and then local champions. The frequency of trainings increased first after the introduction of motivating champions. Time from birth to first ventilation decreased; still, merely 16% of newborns received ventilation within the first minute as recommended. This is a remaining challenge that may require more targeted team-scenario training and quality improvement efforts to improve.

**Supplementary Information:**

The online version contains supplementary material available at 10.1186/s41077-022-00234-z.

## Background

Annually, 1.5 million fresh stillbirths (FSB) and early newborn deaths occur due to intrapartum-related events [1, 2]. The majority of these deaths, including misclassified FSB, could be prevented if non-breathing newborns received skilled resuscitation at birth [3]. Helping Babies Breathe (HBB) is a simulation-based educational program, implemented in more than 80 low-resourced countries, with the aim of increasing skills among birth attendants in newborn resuscitation [4]. The program is shown to be cost-effective, able to enhance knowledge and skills, and able to reduce perinatal mortality [5]. HBB 2nd Edition was launched in 2016, emphasizing educational advice, program implementation, and strategies for quality improvement [6]. Simultaneously, a new and improved simulator, NeoNatalie Live (Laerdal Global Health, Stavanger, Norway), was developed and has shown to provide realistic training comparable to real-life resuscitations [7, 8].

Previous studies have found low-dose high-frequency training strategies effective for knowledge and skills retention [5, 9, 10]. However, individual studies show varied results regarding the translation of improved training skills to clinical practice [5, 11–14]. Two systematic reviews show no overall difference in drying, stimulation, suctioning, or bag-mask ventilation (BMV) after HBB implementation [15, 16]. The number of newborns receiving BMV within 60 s increased significantly in one study following a quality improvement cycle for HBB implementation at a tertiary hospital in Nepal [17]. A study from a high-resourced setting, testing the NeoSim simulation-based training (SBT), demonstrated reduced mortality, and decreased need for suction, BMV, intubation, chest compressions, and adrenaline during newborn resuscitation [18].

In real-life newborn resuscitations, a most crucial step is to start ventilation within the “golden minute” after birth [19, 20]. Preparedness, including available resuscitation equipment, is a key factor for successful resuscitations [19, 21]. Mastery of BMV skills, both during training and in real-life resuscitations, are challenging [22, 23]. Well-known barriers to training include staff turnover and limited time [5, 24, 25]. The rationale for the present study was the need for more research on how to implement and sustain frequent training and improve the translation of training skills into clinical practice.

HBB 2nd Edition, along with the new simulator, NeoNatalie Live, was implemented at Haydom Lutheran Hospital, in Tanzania in September 2016 [26]. The implementation consisted of two phases: first, an intervention period of mainly self-guided individual skill training (13 months) and second, individual skill training and scenario team training facilitated by local champions (11 months). During the first intervention period, 688 skill trainings and 40 team trainings were conducted, whereas 8451 skill trainings and 307 team trainings were conducted during the second intervention period, with a significant increase in staff participation and training performance after the appointment of local champions [26].

The aim of the present study was to describe changes in clinical resuscitation performance and perinatal outcomes, before and after the introduction of the novel simulator (in September 2016) and then local champions (in October 2017) to facilitate ongoing in situ HBB skills and scenario SBT among midwives.

## Methods and material

This is a prospective observational study from September 1, 2015, through August 31, 2018, at Haydom Lutheran Hospital in rural Tanzania. All deliveries were observed by research assistants, and all live births and FSB were included in the analysis. The study was approved by the National Institute for Medical Research, Tanzania (NIMR/HQ/R.8a/Vol.IX/2877) and the Regional Committee for Medical and Health Research Ethics, Western Norway (Ref.no. 2013/110). Informed consent from the mothers was not required by the ethical committees due to the descriptive quality improvement study design.

### Study setting and participants

**Fig. 1:**
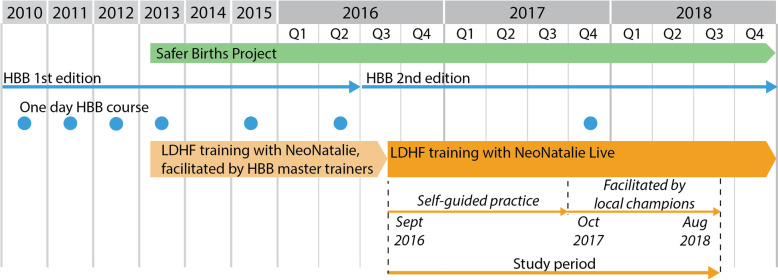
SBT at Haydom Lutheran Hospital 2010–2018. Simulation-based training at Haydom Lutheran Hospital 2010–2018. HBB, Helping Babies Breathe; LDHF, low-dose high-frequency training; local champions, dedicated midwives in charge of facilitation and motivation for one-site training in HBB 2nd Edition. Figure by Ingunn Anda Haug (Laerdal Medical) [26]

Haydom is a rural referral hospital in Northern Tanzania that serves a catchment area of approximately 2 million people and has 3500–4000 deliveries annually. Deliveries and newborn resuscitations are mainly conducted by 18–22 midwives, with physicians on-call. The hospital has emphasized SBT since the introduction of HBB 1st Edition in 2010 and further through the Safer Births project as described by Mduma et al. [12]. An overview of SBT at Haydom, prior to and throughout this study period, is presented in Fig. 1. Simulations were conducted by midwives working in the maternity ward. The number of midwives varied throughout the study period due to a high staff turnover (*n*=15–27). Other healthcare workers, i.e., physicians and students, were allowed to use the simulator, but were not part of the SBT intervention.

### Interventions

#### NeoNatalie Live—newborn resuscitation simulator

NeoNatalie Live is a substantially improved newborn resuscitation simulator, compared to the original NeoNatalie (Laerdal Global Health, Stavanger, Norway) and provides both individual skill training and scenario team training. The learner guides for the simulator are available in the supplementary information (Additional files 1 and 2). The simulator features variable lung compliance, realistic BMV training, and four different patient cases [8]. The simulator provides automated feedback based on HBB guidelines. The presented feedback is prioritized after the following lists: missing head tilt, insufficient opening ventilations, paused ventilations, mask leak, too high-ventilation pressure, and ventilation rate.

#### Implementation of NeoNatalie Live and local champions

Preceding implementation, we observed a 1-year baseline period (period 1) with low-dose high-frequency HBB training using the original NeoNatalie simulator. During the first intervention phase (period 2), September 1, 2016–September 30, 2017, Haydom continued HBB training using the 2nd Edition and introduced NeoNatalie Live in their labor ward (Fig. 1). The improved simulator was mainly used for individual skill training according to self-guided practice, and 688 skill-trainings were conducted in this period 2 [26]. In addition, 40 scenario team trainings were performed in the same period.

Initiating the second intervention phase (period 3), October 1, 2017–August 31, 2018, the hospital management appointed four dedicated midwives, “local champions,” to facilitate and motivate for training. The local champions were junior midwives, carefully selected according to their personality traits and their beneficial influence in the ward. They were responsible for encouraging midwives to train and for technical support to ensure an all-time functioning simulator. They were tasked to arrange scenario team training and motivate the midwives to conduct self-guided skill training whenever time allowed. The local champions received a Master Trainer Guide and a Learner Guide for the simulator (Additional files 1 and 2) preceding the appointment. They did not receive any remuneration for this specific assignment or possess other roles apart from their clinical tasks. In period 3, a total of 8451 skill trainings and 307 scenario team trainings were conducted [26].

### Data collection and management

Trained research assistants, working in 3 shifts, 24 h a day, observed and recorded information (labor and newborn characteristics, events, and resuscitation interventions) on a data collection form. Trained data clerks double-entered data using Epidata 3.1. Biomedical signal data during resuscitations from September 1, 2015, until June 26, 2018, were obtained using the Newborn Resuscitation Monitor (Laerdal Global Health, Stavanger, Norway). The monitor had sensors to synchronously capture and record heart rate from ECG, as well as airway pressure, flow, volume, and expired CO_2_ during BMV using a self-inflating bag [27]. All sensors were located between the resuscitator bag and the facemask.

### Outcomes

The main outcomes were the proportion of newborns receiving stimulation, suction, and/or BMV immediately after birth and time from birth to first ventilation. In addition, the total duration of BMV, pauses during BMV (pauses >3 s), ventilation frequency, tidal volumes, and mask leak was observed. We also investigated initial newborn heart rate, increase in newborn heart rate during BMV, and frequency of BMV training. Secondary outcomes included FSB, a 24-h neonatal mortality, and admissions to the Neonatal Care Unit.

### Statistical methods

Data analysis was performed by Matlab R2021a (MathWorks, Natick, MA) and SPSS version 26 (IBM Corp., Armonk, NY). Continuous data were presented by mean±SD or median (quartiles) and categorical data by percentages (numbers), unless otherwise stated. To test for differences over the periods in continuous variables, Kruskal-Wallis test was used. If a significant difference over the periods was found, this was further examined by pairwise comparisons between the periods, using the Mann-Whitney test and a Bonferroni adjustment for multiple testing. For categorical data, the same approach was used, but then applying the chi-squared or Fischer’s exact test was appropriate. Logistic regression was used to test for differences between the periods in proportions of fatal outcomes adjusted for risk factors. A significance level of 0.05 was used in all hypothesis tests.

## Results

During the study period, 10,672 births were recorded. The 191 macerated stillbirths (i.e., suspected dead before onset of labor) were excluded, while 10,481 births, including 135 FSB, were included for further analysis. Throughout the study period, 816 newborns received BMV and 644 of these resuscitations were recorded using the Newborn Resuscitation Monitor. Resuscitations were mainly performed by midwives, and some were provided by physicians or students. There were 12% missing registrations for the variable “cervical dilatation” and <0.1% for the remaining variables.

### Patient vulnerability and perinatal outcome

The proportion of high-risk deliveries increased during the study period. From baseline to each intervention period, a higher number of admissions came from health centers (referrals) and the frequency of abnormal fetal heart rate on admission and during labor increased (Table 1). The proportion of premature newborns increased significantly, from 2.9 to 4.1%, corresponding to an increase in newborns with low birth weight <1500 g (Table 2).

**Table 1:** Labor and maternal characteristics of live births and fresh stillbirths (*n*=10,481). Values are given as mean± SD or percentage (number)

	Period 1 Baseline	Period 2 Individual skill training	Period 3 Local champions	***P*** value	Period 1 vs 2	Period 1 vs 3	Period 2 vs 3
	01.09.15–31.08.16	01.09.16–30.09.17	01.10.17–31.08.18				
**Months** (*n*)	12	13	11				
**Births** (*n*)	3572	4003	2 906				
**Admission from the health center**	3.9 (140)	5.6 (226)	5.9 (171)	<0.001^a^	<0.001^a^	<0.001^a^	0.675^a^
**Mother had no formal education**^**b**^	9.3 (196)	8.2 (328)	6.3 (184)	<0.001^a^	0.140^a^	<0.001^a^	<0.004^a^
**Maternal age**^**b**^	26.2 ±6.9	26.1 ±6.7	26.1 ±6.8	0.835^c^			
**No antenatal care**	0.9 (33)	0.9 (35)	1.0 (28)	0.928^a^			
**Antenatal problem**	1.5 (52)	2.0 (79)	2.3 (67)	0.039^a^	0.084^a^	0.011^a^	0.343^a^
**Cervical dilatation on admission** (cm)	5.7 ±2.5	5.6 ±2.6	5.8 ±2.6	0.009^c^	0.897^d^	0.009^d^	0.005^d^
**10-cm cervical dilation on admission**	6.7 (212)	7.4 (262)	8.5 (212)	0.044^a^	0.263^a^	0.013^a^	0.135^a^
**Hours in labor before admission** (h:min)	5:08 ±2:29	4:28 ±2:32	4:44 ±2:15	<0.001^c^	<0.001^d^	<0.001^d^	0.004^d^
**Fetal heart rate on admission**				0.001^a^	0.001^a^	0.006^a^	0.744^a^
Normal (120–160 bpm)	91.7 (3274)	93.1 (3724)	93.2 (2709)				
Abnormal (<120 or >160)	1.0 (37)	1.2 (49)	1.3 (38)				
Not detectable	0.5 (19)	0.9 (37)	0.7 (20)				
Not measured	6.7 (241)	4.8 (192)	4.8 (139)				
**Fetal heart rate during labor**				<0.001^a^	<0.001^a^	<0.001^a^	0.022^e^
Normal (120–160 bpm)	82.6 (2952)	85.5 (3423)	85.5 (2486)				
Abnormal (<120 or >160)	5.3 (190)	7.3 (291)	8.7 (252)				
Not detectable	0.5 (19)	1.0 (40)	0.7 (20)				
Not measured	11.5 (411)	6.2 (248)	5.1 (148)				
**Final fetal heart rate before delivery**	134.0 ±14.0	132.9 ±17.3	132.5 ±17.3	0.075^c^			
**Mode of delivery**				0.020^a^	0.181^a^	0.222^a^	0.010^a^
Spontaneous vaginal delivery	75.3 (2690)	75.8 (3035)	75.0 (2179)				
Cesarian delivery	23.8 (851)	23.0 (922)	23.7 (690)				
Assisted breech delivery	0.6 (22)	0.5 (22)	1.1 (31)				
Vacuum extraction	0.3 (9)	0.6 (23)	0.2 (5)				
Others	0.0 (0)	0.0 (1)	0.0 (1)				
**Singleton/multiple**				0.353^a^			
Singleton	96.0 (3428)	96.1 (3847)	96.6 (2808)				
Multiple	4.0 (144)	3.9 (56)	3.4 (98)				
**Labor complications**^**f**^	2.4 (85)	2.3 (94)	2.7 (78)	0.634^a^			
Uterine rupture	0.2 (6)	0.1 (5)	0.1 (3)	0.763^a^			
Pre-eclampsia	0.5 (17)	0.6 (25)	0.6 (16)	0.686^a^			
Eclampsia	0.3 (9)	0.2 (7)	0.5 (14)	0.056^a^			
Cord prolapse	0.8 (25)	0.7 (29)	0.6 (16)	0.653^a^			
Prepartum bleeding	0.7 (23)	0.8 (31)	1.0 (28)	0.348^a^			
Shoulder dystocia	0.2 (7)	0.0 (1)	0.1 (3)	0.062^g^			

^a^Chi-squared test

^b^Data collection initiated 01.02.2016

^c^Kruskal-Wallis test

^d^Mann-Whitney test

^e^Not significant after Bonferroni correction

^f^One or several labor complications

^g^Fischer’s exact test

**Table 2:** Newborn characteristics, resuscitation characteristics, and outcome of live births and fresh stillbirths (*n*= 10,481). Values are given as mean± SD or percentage (number)

	Period 1 Baseline	Period 2 Individual skill training	Period 3 Local champions	***P*** value	Period 1 vs 2	Period 1 vs 3	Period 2 vs 3
	01.09.15–31.08.16	01.09.16–30.09.17	01.10.17–31.08.18				
**Births** (*n*)	3572	4003	2906				
**Newborn characteristics**							
**Gestational age** (week)	38.8 ±1.9	38.7 ±1.9	38.6 ±2.0	0.003^a^	0.620^b^	0.001^b^	0.005^b^
**Preterm**	2.9 (103)	3.8 (151)	4.1 (118)	0.024^c^	0.032^cd^	0.009^c^	0.540^c^
**Birth weight** (gram)	3302.4 ±523.6	3357.7 ±556.6	3335.3 ±550.9	<0.001^a^	<0.001^b^	0.002^b^	0.115^b^
**Birth weight categories** (gram)				<0.001^c^	<0.001^c^	0.002^c^	0.038^cd^
<1500	0.5 (18)	0.8 (32)	1.0 (30)				
1500–2499	5.0 (178)	4.9 (195)	4.3 (126)				
2500–3499	56.2 (2008)	50.2 (2011)	52.7 (1531)				
3500–4499	37.2 (1328)	42.2 (1690)	40.2 (1167)				
≥4500	1.1 (40)	1.9 (75)	1.8 (52)				
**Resuscitation characteristics**							
**Resuscitation kit present**	92.7 (3313)	93.5 (3742)	93.6 (2721)	0.276^c^			
**Bag/mask resuscitator present**	91.0 (3252)	98.1 (3923)	97.8 (2842)	<0.001^c^	<0.001^c^	<0.001^c^	0.456^c^
**Stimulation**	31.0 (1109)	27.6 (1104)	34.4 (1001)	<0.001^c^	0.001^c^	<0.004^c^	<0.001^c^
**Suction**	28.2 (1009)	23.6 (943)	28.0 (815)	<0.001^c^	<0.001^c^	0.857^c^	<0.001^c^
**Bag/mask ventilation**	7.0 (249)	8.0 (319)	8.5 (248)	0.056^c^			
**APGAR 1 min**	8.6 ±1.2	8.5 ±1.5	8.5 ±1.4	0.537^a^			
**APGAR 5 min**	9.8 ±1.2	9.7 ±1.5	9.7 ±1.4	<0.001^a^	0.006^b^	<0.001^b^	0.049^bd^
**Low APGAR 1 min** (APGAR ≤7)	4.0 (143)	5.0 (200)	5.7 (167)	0.005^a^	0.038^ad^	0.001^a^	0.170^a^
**Low APGAR 5 min** (APGAR ≤7)	2.3 (81)	3.9 (157)	4.2 (123)	<0.001^c^	<0.001^c^	<0.001^c^	0.518^c^
**Perinatal outcome at 30 min**				0.013^c^	0.008^c^	0.013^c^	0.563^c^
Normal	93.6 (3345)	91.6 (3666)	91.6 (2662)				
Admitted neonatal unit	5.0 (179)	6.5 (262)	6.9 (200)				
Death	0.3 (9)	0.3 (13)	0.3 (10)				
Fresh stillbirth	1.1 (39)	1.5 (62)	1.2 (34)				
**Neonatal outcome at 24 h**				0.003^c^	0.002^c^	0.012^c^	0.261^c^
Normal	95.1 (3396)	93.0 (3723)	93.5 (2715)				
Still in the neonatal care unit	3.2 (114)	4.4 (178)	4.0 (116)				
Death	0.6 (23)	1.0 (40)	1.3 (38)				

Fresh stillbirth is reported for perinatal outcome at 30 min

^a^Kruskal-Wallis test

^b^Mann-Whitney test

^c^Chi-square test

^d^Not significant after Bonferroni correction

In unadjusted analyses, perinatal mortality (i.e., 24-h newborn deaths and FSB) increased slightly during the study period (*p* value 0.038). After adjustment for the source of admission, premature/term, birth weight, mode of delivery, and the last fetal heart rate before delivery, there was no significant change in risk for perinatal deaths (*p* value 0.898).

### Changes in resuscitation performance and training frequency

The proportion of births with a bag/mask resuscitator present before the delivery increased from 91.0 to 98.1% (Table 2). The observed proportion of newborns receiving BMV increased over the periods, from 7.0 to 8.5%, although not statistically significant (*p* value 0.056) (Table 2). We observed a significant decrease in both suction and stimulation in period 2 compared to period 1, but an increase in stimulation between periods 2 and 3 (Table 2). The median time from birth to the start of ventilation decreased from 118 to 101 s (*p* value 0.007) over the whole study period (Table 3). Resuscitations were performed by midwives in 66.3% of the cases in period 1 vs 77.1% in period 2 and 83.5% in period 3 (Table 3). The proportion of midwives who had trained, using NeoNatalie Live, during the last 7 days prior to the resuscitation, increased from 33.8% in period 1 to 71.4% in period 3 (Table 3).

**Table 3:** Resuscitation characteristics of newborns receiving bag/mask ventilation (*n*=816). Values are given as median (quartiles) or percentage (number)

	Period 1 Baseline	Period 2 Individual skill training	Period 3 Local champions	***P*** value	Period 1 vs 2	Period 1 vs 3	Period 2 vs 3
	01.09.15–31.08.16	01.09.16–30.09.17	01.10.17–31.08.18				
**Newborns receiving BMV** (*n*)	249	319	248				
**Midwife providing resuscitation**	66.3 (165)	77.1 (246)	83.5 (207)	<0.001^a^	0.004^a^	<0.001^a^	0.061^a^
**Midwife trained with NeoNatalie last 7 days**	33.8 (54)	29.6 (72)	71.4 (147)	<0.001^a^	0.383^a^	<0.001^a^	<0.001^a^
**Time from birth to the start of BMV** (seconds)	118 (85–165)	100 (74–160)	101 (72–150)	0.018^b^	0.028^cd^	0.007^c^	0.532^c^
**BMV initiated within 60 s**	13.3 (33)	13.2 (42)	15.7 (39)	0.633^a^			
**Perinatal outcome at 30 min**				0.213^a^			
Normal	58.2 (145)	51.7 (165)	52 (129)				
Admitted neonatal unit	33.3 (83)	42.3 (135)	42.3 (105)				
Death	3.2 (8)	3.4 (11)	2.4 (6)				
Fresh stillbirth	5.2 (13)	2.5 (8)	3.2 (8)				
**Neonatal outcome at 24 h**				0.398^a^			
Normal	66.7 (166)	62.4 (199)	62.6 (154)				
Still in the neonatal care unit	21.7 (54)	25.7 (82)	24.8 (61)				
Death	6.4 (16)	9.4 (30)	9.3 (23)				

*BMV* bag/mask ventilation

^a^Chi-square test

^b^Kruskal-Wallis test

^c^Mann-Whitney test

^d^Not significant after Bonferroni correction

There was a significant decrease in time to start BMV, but no significant change in newborns being ventilated within the golden first minute from birth or the time of applied BMV, throughout the study period. The proportion of pauses during ventilation decreased from 27.9 to 15.7%, and ventilation frequency increased slightly from 45 to 48 ventilations per/min (Table 4). There was no statistically significant change in mask leak, but expired tidal volume decreased from 7.6 to 6.3 ml/kg (Table 4). Static newborn lung compliance decreased during the whole study period (Table 4).

**Table 4:** Bag/mask ventilation characteristics recorded from the newborn resuscitation monitor (*n*=644). Values are given as median (quartiles) or percentage (number)

	Period 1 Baseline	Period 2 Individual skill training	Period 3 Local champions	***P*** value	Period 1 vs 2	Period 1 vs 3	Period 2 vs 3
	01.09.15–31.08.16	01.09.16–30.09.17	01.10.17–26.06.18				
	220	268	156				
**Heart rate characteristics**							
First HR (bpm)	98 (61–152)	95 (62–149)	88 (60–140)	0.699^a^			
First HR under 100 bpm (%)	50.9 (112)	50.7 (136)	55.1 (86)	0.644^a^			
HR after last ventilation (bpm)	153 (130, 171)	157 (134, 169)	160 (137, 170)	0.621^a^			
HR 60 s after last ventilation (bpm)	160 (138, 176)	163 (145, 176)	160 (140, 175)	0.550^a^			
**BMV characteristics**							
Total BMV duration (seconds)	135 (66, 349)	127 (64, 266)	152 (82, 347)	0.234^a^			
Total BMV pause (%)	27.9 (15.2, 44.8)	19.1 (8.2, 34.9)	15.7 (4.0, 31.2)	<0.001^a^	<0.001^b^	<0.001^b^	0.077^b^
Tidal volume expired, (ml/kg)	7.6 (4.8, 11.8)	6.0 (2.9, 9.8)	6.3 (3.5, 10.4)	<0.001^a^	<0.001^b^	0.025^bc^	0.137^b^
Mask leak (%)	38.0 (15.8, 57.0)	42.0 (18.5, 61.8)	38.8 (16.0, 60.0)	0.495^a^			
Ventilation frequency, (1/min)	45.0 (36.0, 55.0)	48.0 (42.0, 54.0)	48.0 (42.2, 54.0)	0.006^a^	0.009^b^	0.005^b^	0.542^b^
Peak inspiratory pressure (mbar)	36.0 (30.0, 39.0)	39.0 (36.0, 40.0)	38.0 (37.0, 40.0)	<0.001^a^	<0.001^b^	<0.001^b^	0.498^b^
Minute volume (ml)	369 (225, 556)	287 (181, 459)	324 (198, 449)	0.005^a^	0.001^b^	0.049^bc^	0.335^b^
Number of ventilations	61 (34, 159)	72 (38, 147)	85 (44, 208)	0.017^a^	0.348^b^	0.006^b^	0.029^bc^
Static lung compliance, (ml/mbar)	0.93 (0.60, 1.40)	0.64 (0.38, 1.01)	0.67 (0.40, 1.02)	<0.001^a^	<0.001^b^	<0.001^b^	0.630^b^
**First minute of ventilation**							
Total BMV pause during first minute (%)	31.1 (12.3, 56.6)	23.6 (8.3, 42.1)	15.1 (5.5, 38.7)	<0.001^a^	0.003^b^	<0.001^b^	0.020^bc^
Tidal volume expired (ml/kg)	7.0 (3.5, 10.6)	4.2 (1.4, 8.1)	4.3 (1.9, 8.7)	<0.001^a^	<0.001^b^	<0.001^b^	0.443^b^
Mask leak (%)	42.2 (21.0, 68.2)	52.8 (24.2, 74.5)	52.5 (26.0, 72.0)	0.068^a^			
Ventilation frequency, (1/min)	46.0 (37.0, 58.0)	48.0 (42.0, 55.5)	49.0 (43.0, 55.0)	0.058^a^			
Peak inspiratory pressure (mbar)	37.0 (32.0, 40.0)	39.0 (37.0, 41.0)	38.8 (37.0, 41.5)	<0.001^a^	<0.001^b^	<0.001^b^	0.742^b^
Minute volume (ml)	317 (183, 494)	241 (113, 385)	247 (153, 387)	<0.001^a^	<0.001^b^	0.002^b^	0.500^b^
Number of ventilations	30 (20, 40)	36 (27, 44)	39 (29, 44)	<0.001^a^	<0.001^b^	<0.001^b^	0.190^b^
Static lung compliance (ml/mbar)	0.93 (0.61, 1.41)	0.65 (0.39, 1.04)	0.71 (0.41, 1.06)	<0.001^a^	<0.001^b^	<0.001^b^	0.396^b^

*HR* heart rate, *BMV* bag/mask ventilation, *Bpm* beat per minute

^a^Kruskal-Wallis test

^b^Mann-Whitney test

^c^Not significant after Bonferroni correction

## Discussion

Following a period of frequent in situ SBT in newborn resuscitation, preparedness for resuscitation and the number of newborns receiving BMV increased. Time to first ventilation and pauses during ventilation decreased significantly. The vulnerability among admitted women and their newborns seemed to increase during the study period. In line with the intention, we found a significant increase in self-reported training using NeoNatalie Live over the last 7 days before conducting a real resuscitation, from 33.8 to 71.4% throughout the study period, and midwives performed real resuscitations more frequently during the intervention periods.

The use of stimulation during real resuscitation decreased from period 1 to period 2 but increased from period 1 to period 3. Stimulation is an initial intervention in newborn resuscitation and marks the initiation of resuscitation, thus reflecting the number of newborns receiving resuscitation. The increased use of stimulation may reflect the more vulnerable population of newborns or increased training. We documented a decrease in the use of the suction from period 1 to period 2; still, there were no changes from period 1 to period 3. Two systematic reviews found no change in the frequency of either stimulation or suction after HBB training [15, 16]. Newborn resuscitation guidelines no longer recommend suction unless the airway is obstructed by secretions, and several studies show decreased use of unnecessary suctioning after HBB training [17, 19, 28].

As much as 8.5% of the newborns received BMV in period 3, compared to 7.0% during period 1. Several individual studies report more frequent BMV following the implementation of HBB [17, 28, 29]. However, two systematic reviews found no change in the frequency of BMV after HBB training [15, 16]. The not significant tendency towards more newborns receiving BMV in our study is likely due to a more vulnerable population with an increased number of newborns requiring BMV. Daily simulation-based training during the second intervention period might also have contributed to enhanced confidence and thus increased use of BMV. The aim of HBB training is to provide timely and skilled BMV when needed. The actual proportion of non-breathing newborns who would benefit from BMV is probably unknown and varies across settings, depending on maternal and newborn vulnerability and obstetric and newborn care. Thus, it may be difficult to evaluate if the observed frequency of BMV is appropriate and to compare it with other settings. Importantly, all newborns being ventilated were observed by the trained research assistants as “not breathing” until the start of BMV.

Rapid initiation of BMV among non-breathing newborns is critical for intact survival, and guidelines recommend the start of ventilation within 1 min after birth [19, 20]. Time from birth to first ventilation decreased from 118 to 101 s during our study period. Still, merely 15.7% of the newborns receiving BMV were ventilated within the first minute of life, confirming previous literature [28, 30]. Chaulagain et al. found a monthly cumulative median time to first ventilation of 153 s and a proportion of 11.1% of the newborns ventilated within the first minute, showing that even after a targeted quality improvement package in newborn resuscitation, rapid initiation of BMV remains a great challenge [28]. In our study, the increase in births with bag/mask resuscitators present prior to delivery, demonstrating an increased preparedness for resuscitation, in addition to increased skills might have impacted the time to start ventilation [31, 32].

Over the study period, midwives at Haydom conducted a large amount of both individual skill training [26] and organized scenario team trainings. It is not possible to analyze how these different training modalities separately may have influenced the documented changes in clinical care. However, we believe that scenario team-trainings may typically influence team work, decision making, and clinical treatment timelines more than individual skill training would do [33, 34]. Therefore, we speculate that more targeted team-scenario trainings in combination with specific continuous quality improvement efforts are necessary to ensure that most non-breathing newborns receive BMV within the first minute.

Regarding ventilation performance, healthcare workers managed to ventilate with less pauses after increased use of self-guided skill training in period 2. The simulator provides feedback regarding this specific action point in the HBB algorithm, making it reasonable that training contributed to enhancing this clinical skill. The observed reduction in expired tidal volume and minute volume during the study period are most likely due to a more vulnerable newborn population. This is supported by the findings of decreased static lung compliance indicating stiffer lungs and thus increased difficulties in providing a sufficient minute volume during BMV, despite the increased number of ventilations and peak inspiratory pressure. Low lung compliance likely represents unmature preterm lungs and/or liquid-filled lungs of severely asphyxiated newborns. To establish functional residual capacity in such lungs may be difficult and likely explains the almost significant trend towards more mask leaks in the first minute of BMV [35].

Regarding mortality, overall 30-min newborn mortality seems to be stable around 3/1000 and the FSB rate changes from 1.1 to 1.5 % throughout the study period, despite an increased population vulnerability (Table 2). The 30-min perinatal outcome is typically associated with intrapartum-related events and complications, causing FSB or severely asphyxiated newborns [36]. The 24-h newborn mortality mainly reflects intrapartum-related deaths, but may also include some deaths secondary to other causes, e.g., prematurity and severe infections [37]. SBT interventions combining obstetric and newborn care might be needed for reducing perinatal mortality [33, 38]. The increase in more vulnerable women being admitted over time is difficult to explain based on our data alone. However, a recent paper from the same setting describes how this poor catchment population reacts to changing conditions like introducing patient fees for ambulance service and hospital delivery [39].

Among several strengths of this study is the large population size. In addition, the rigorous data management system, comprehensive data collection form presenting detailed information on resuscitation practice, combined with physiological data during the first minutes of life provides unique information. One limitation is the non-randomized design and thus the inability to claim causality. In addition, lack of qualitative data restricts the interpretation of reasons behind the successes and potential barriers. A mixed method design could have added important knowledge for further improvement of training approaches and eventually clinical performance.

HBB and the new NeoNatalie Live simulator is a low-cost program and thus possible to scale up in other resource-limited settings [40]. However, Haydom has emphasized individual simulation-based HBB skill training for a decade, thus the management and staff are used to such learning methods. This background likely explains their eagerness to use the new simulator, and furthermore, the huge training load response when the local champions started their work. These responses happened despite the high turnover in clinical staff. Settings with less SBT practice may need more time to achieve similar results. High-income settings might require additional training on more advanced interventions for newborn resuscitation, but the implementation strategy of using local champions is considered generalizable.

## Conclusion

Even when caring for a more vulnerable maternal and newborn population, midwives at Haydom managed to improve their clinical skills in newborn resuscitation after an impressive increase in individual training frequency and scenario team trainings using a novel newborn simulator, stimulated by local champions. The low number of newborns receiving BMV within the first minute is a remaining critical challenge, and we think more additional quality improvement efforts are necessary to achieve guideline adherence. Further research should focus on qualitative and/or mixed-methods studies exploring underlying reasons for the described changes and the challenge of timely BMV.

## Supplementary Information

**Additional file 1.** Master Trainer Guide. Information folder for master trainers (local champions) about how to prepare the equipment and perform ventilation training.

**Additional file 2.** Learner Guide. A simple introduction folder for midwives about how to perform ventilation skill training.

## Data Availability

The datasets used and/or analyzed during the current study are available from the corresponding author on reasonable request.

## Abbreviations

BMV: Bag-mask ventilation
FSB: Fresh stillbirths
HBB: Helping Babies Breathe
SBT: Simulation-based skill training
