# Supplementary material for: Newborn resuscitation simulation training and changes in clinical performance and perinatal outcomes: a clinical observational study of 10,481 births
Source: Adv Simul (Lond). 2022 Nov 5;7:38. doi: 10.1186/s41077-022-00234-z (PMC9636744; doi:10.1186/s41077-022-00234-z)
Supplement: Supplementary file 1 — Additional file 1. Master Trainer Guide. Information folder for master trainers (local champions) about how to prepare the equipment and perform ventilation training. [file 41077_2022_234_MOESM1_ESM.pdf]

Newborn Ventilation Trainer

Master Trainer Guide

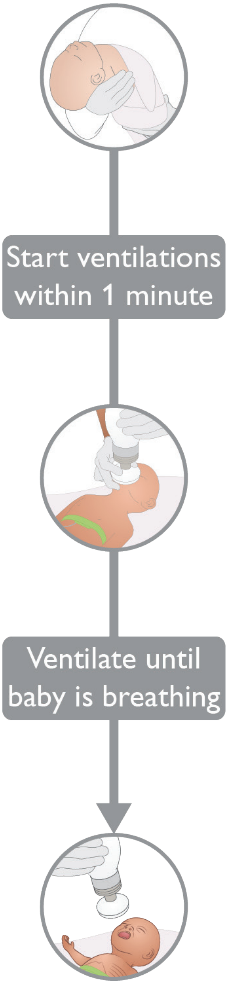

Laerdal
helping save lives

To Master Trainers at Haydom Lutheran Hospital,

Congratulations with the Newborn Ventilation Trainer, *a result of the close collaboration between midwives, doctors, researchers and product developers in Tanzania and Norway in the Safer Births project.*

Focus in the newborn ventilation training is quick initiation of ventilations, continuous ventilation and effective ventilations.

We hope master trainers and midwives enjoy the product and that everybody becomes more proficent with repeated training.

Good luck!

Ingunn Anda Haug and Øystein Gomo

Overview	4
Introduction	5
Startup and Charging	8
Registration of Learners	10
How the Manikin works	12
Training Levels and Learning Objectives	14
How to do Ventilation Skill Training	16
How to do Ventilation Scenario Training	18

Overview

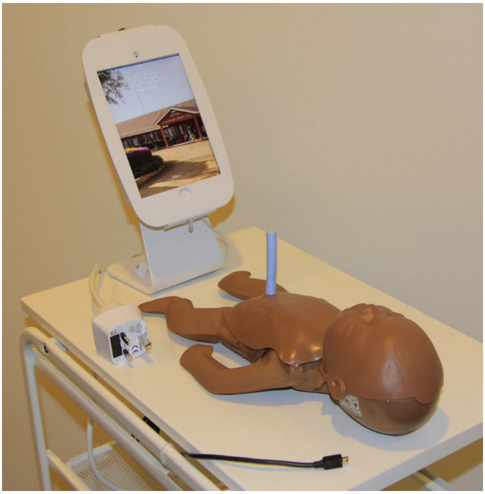

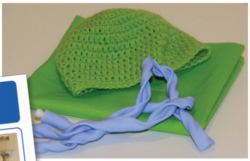

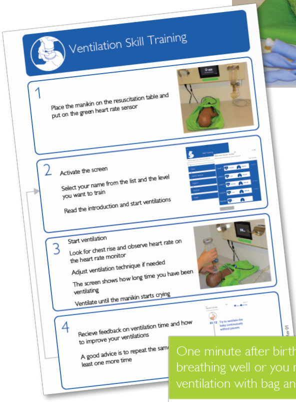

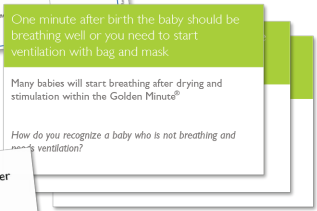

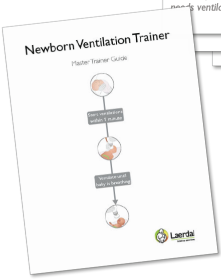

Screen
The screen is used to register training, select training level and give feedback. The screen is fixed to the trolley.

Manikin
Manikin for ventilation training. To be used with bag/mask and heart rate sensor.

Trolley
Trolley for storage of training equipment. Power cable and manikin charge cable are attached to the trolley.

Hat, blanket and cord
Hat, blanket and cord for scenario training.

Learners Guide
Simple introduction to midwives about how to perform Ventilation Skill Training.

Discussion cards
Discussion points related to newborn resuscitation. The cards can be used in group discussions guided by Master Trainers or other skilled staff.

Master Trainer Guide (this folder)
Information to Master Trainers about how to prepare the equipment and perform ventilation training

Where and when to train

The Newborn Ventilation Trainer should be used in the delivery rooms together with the Laerdal Newborn Resuscitation Monitor.

Learners should be encouraged to practice often. After each training the learners will receive feedback from the screen on how to improve. A good advice is to repeat the same training level at least one more time.

Registration of training

The screen is used to guide and to register all training sessions. Learners will have to be registered by Master Trainers in the learner list before first training.

Training results are sent to a training log. The training log can be viewed from a pc or the screen (password needed):

https://researchsandboxapp.azurewebsites.net/

Master Trainers can use this log to follow up the training activities and invite midwives to regular training.

How to train

The Newborn Ventilation Trainer can be used in two different ways:

- Ventilation Skill Training (individual training - without Master Trainer)
- Ventilation Scenario Training (team training - led by Master Trainer)

Ventilation Skill Training

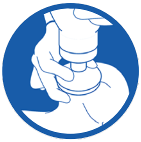

Focus in Ventilation Skill Training is to practice doing **good and continuous ventilations**.

Skill training is for individual learners and can be performed without a master trainer.

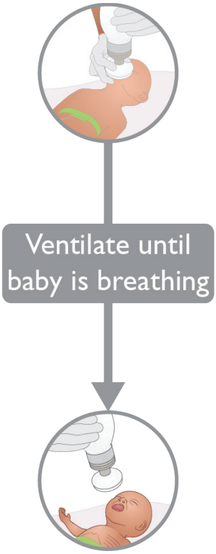

Ventilation Scenario Training

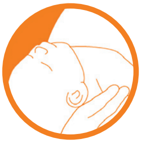

Ventilation Scenario Training means to practice the whole sequence from the baby is born until it is breathing. Focus areas are **The Golden Minute** and **good and continuous ventilations**.

In Scenario Training several learners can practice together. A good advice is to work together as you would during real resuscitations.

Scenario Training is led by a master trainer.

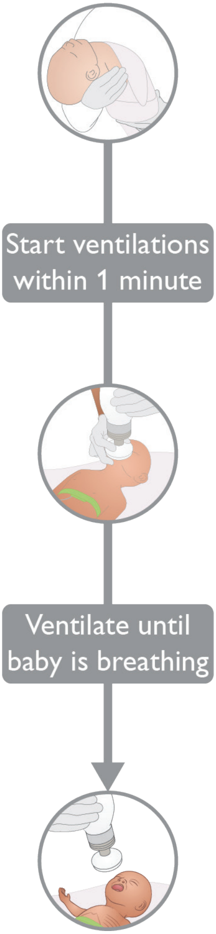

Screen

Press and hold button on upper right corner to turn the screen on

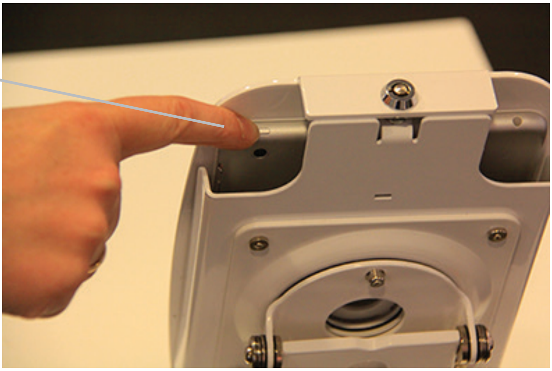

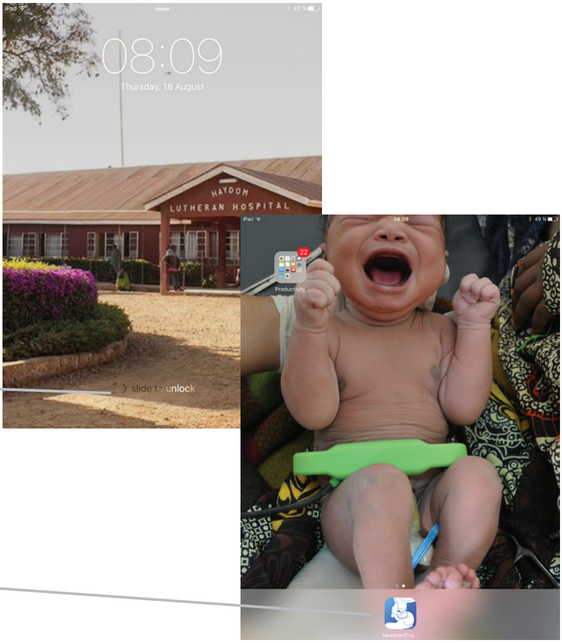

Swipe finger to unlock the screen

Touch 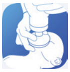 to start Newborn Ventilation Training

To charge the screen, plug the power cable to the wall outlet.

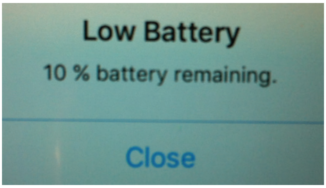

Manikin

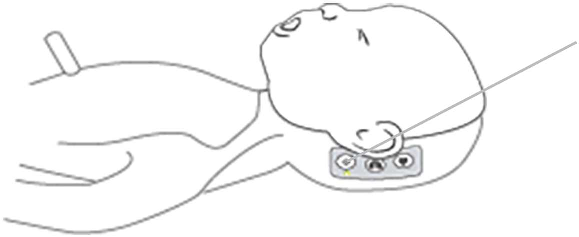

Press and hold 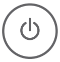 to turn on the manikin.

If no light appears, the manikin must be charged.

The manikin will turn off automatically when it has not been used for a while.

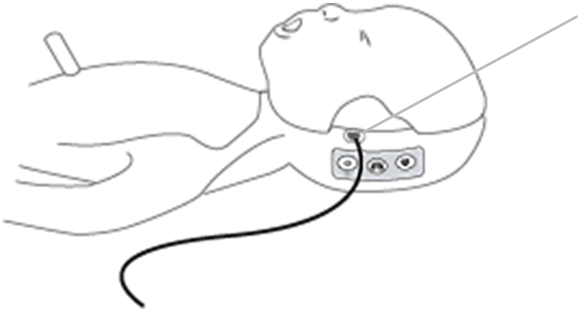

To charge the manikin, plug in the black manikin charge cable under the ear.

Plug the white power cable attached to the trolley to the wall outlet.

Steady light = fully charged and power on

Blinking light = charging

No light = power off or charging needed

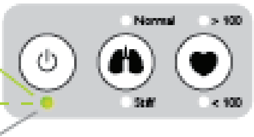

Registration of Learners

The Ventilation Skill Training startup screen shows a list of registered learners. Learners will have to be registered by a master trainer before their first training.

Touch the menu icon
in the upper left corner

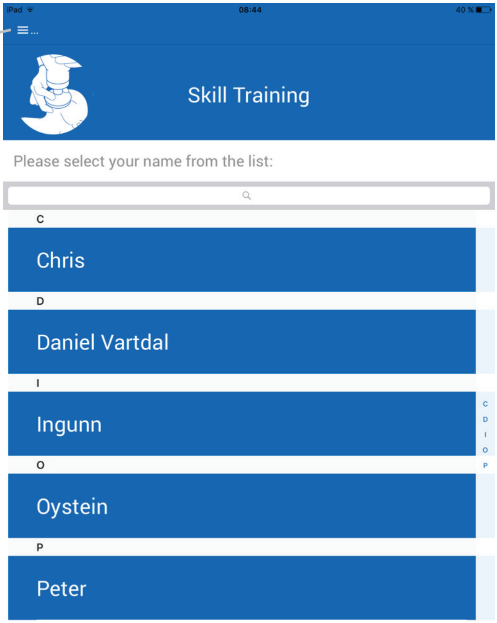

Select "Edit learner list"

You can now add, remove or correct names in the learner list

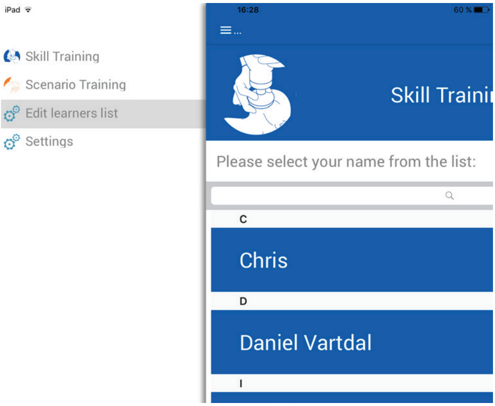

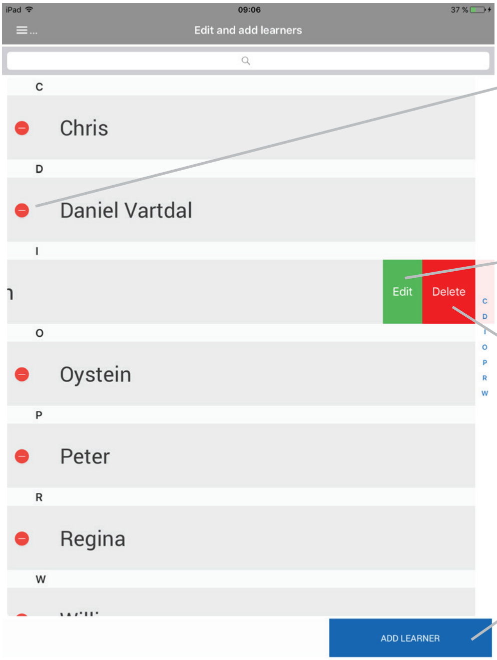

Touch in front of the name you want to edit

Select "Edit" to correct spelling

Select "Delete" to remove the name from the list

Select "Add learner" to include a new learner in the list

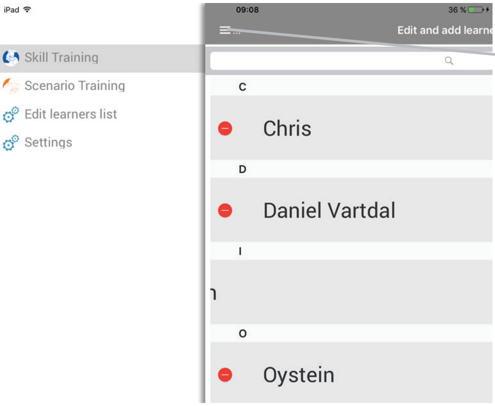

To go back to the startup screen, touch the menu icon in the upper left corner and select "Skill Training" or "Scenario Training"

Ventilations

The manikin represents a newborn baby who is not breathing.
The learners' task is to **ventilate continuously until the baby is breathing.**

To get air into the lungs, you have to

- **open the airways** by tilting the head backwards
- make sure you have a **good mask seal**

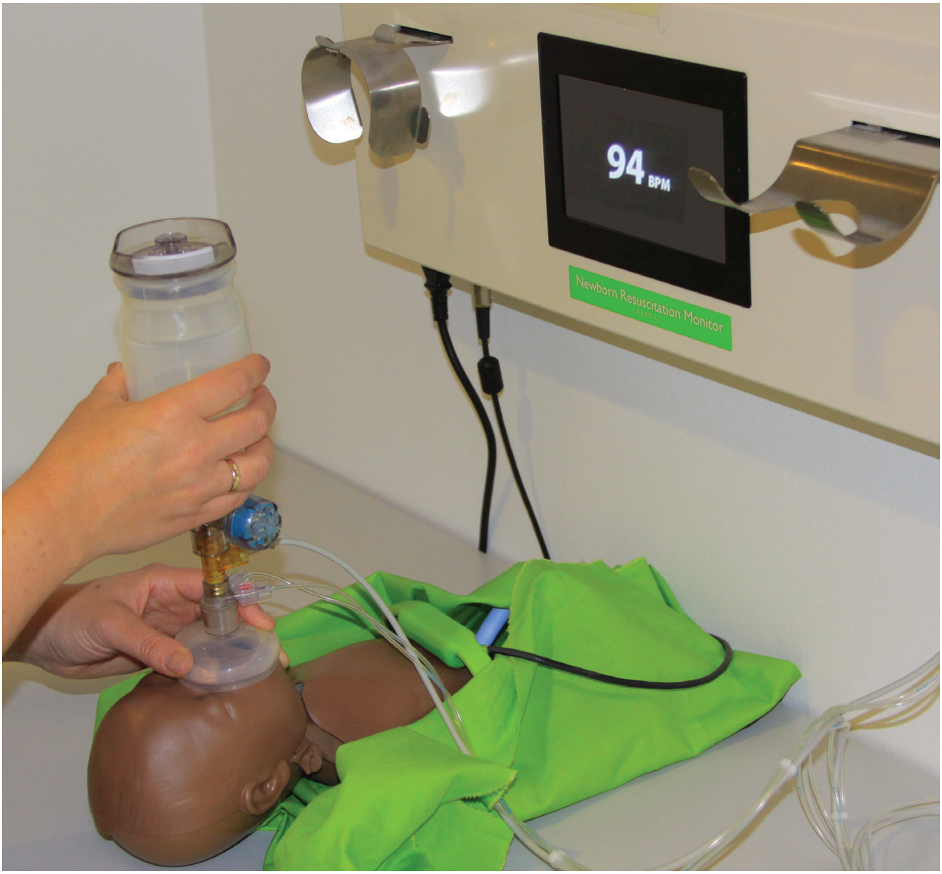

Manikins' response to ventilations

- You will see **chest rise** when the manikin gets air into the lungs
- The **heart rate** will increase with good ventilations and decrease with lack of good ventilations. When the green heart rate sensor is placed on the manikin, the heart rate will be displayed on the monitor on the wall.

  Low heart rate means that the baby has been without oxygen for a while. This may require longer ventilation time. The heart rate will increase with good ventilations.

- The manikin will start **crying** after sufficient time of good ventilations. The crying indicates that the baby is breathing.

Simulation of different patient conditions

Babies who are not breathing may have normal or low heart rate and it may be easy or difficult to get air into the lungs. To simulate this, the manikin has **4 different starting conditions** (Training levels).

Training Levels and Learning Objectives

The Newborn Ventilation Trainer has four different training levels. The main learning objective for all levels is to **ventilate continuously until the baby is breatning**.

- Ventilation technique
  - 40-60 ventilations per minute
  - Head tilt and mask seal needed to get air into the lungs
  - Chest rise indicates that the air enters the lungs

- Relationship between heart rate and ventilations
  - Heart rate will increase with good and continuous ventilations
  - Heart rate will decrease with lack of good and continuous ventilations

Training level 1 to 4 have different heart and lung settings, and represents different patient conditions:

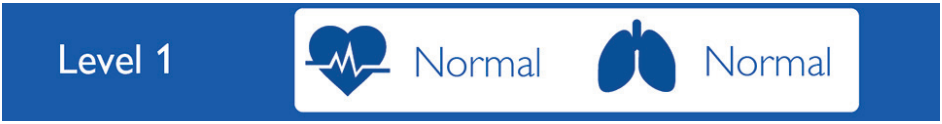

Level 1 represents a baby with initial good heart rate and lungs that are easy to ventilate. Learners should start with this level for **basic ventilation training.**

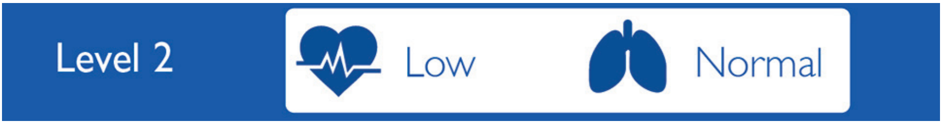

Level 2 represents a baby with initial low heart rate. The learners will see that the **heart rate increases with good and continuous ventilations**.

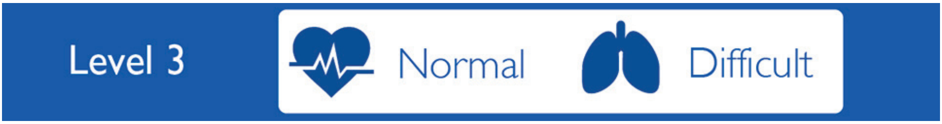

Level 3 represents a baby with initial stiff lungs. The learners have to **give some ventilations with higher pressure to open the lungs**.

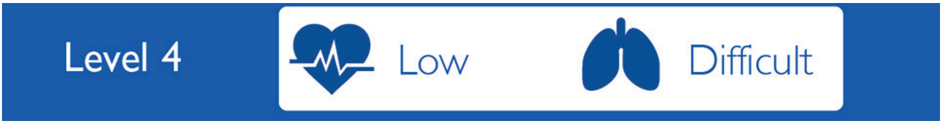

Level 4 represents a baby with both initial low hear rate and stiff lungs. The learners have to **give some ventilations with higher pressure to open the lungs.** When the lungs are open, the learners will see that the **heart rate increases with good and continuous ventilations.**

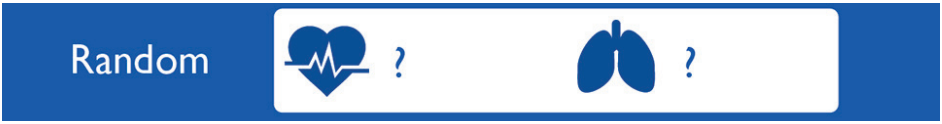

This will be one of the 4 levels above. The learner will not get any information about the patient condition before start.

How to do Ventilation Skill Training

1

Place the manikin on the resuscitation table and put on the green heart rate sensor

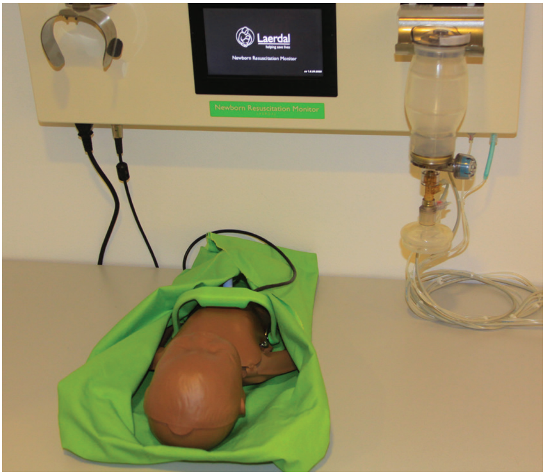

2

Activate the screen

Select your name from the list and the level you want to train

Read the introduction and start ventilations

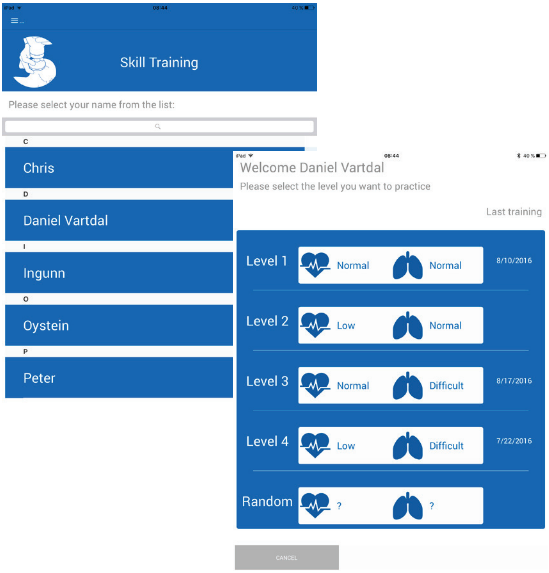

3

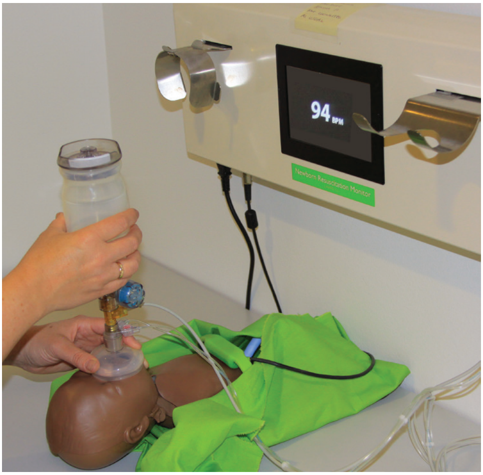

Start ventilation

Look for chest rise and observe heart rate on the heart rate monitor

Adjust ventilation technique if needed

The screen shows how long time you have been ventilating

Ventilate until the manikin starts crying

4

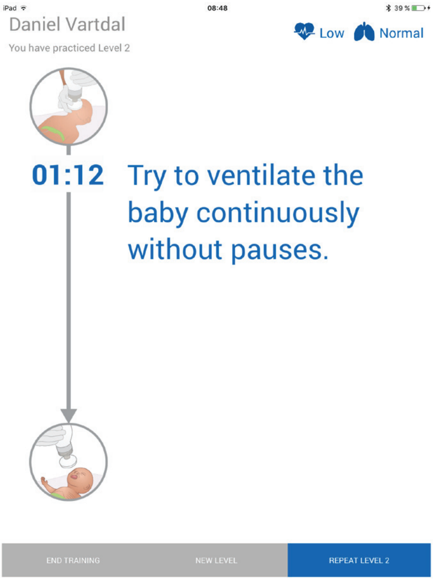

Recieve feedback on ventilation time and how to improve your ventilations

A good advice is to repeat the same level at least one more time

How to do Ventilation Scenario Training

1 - Preparations

Prepare manikin and resuscitation table

- Attach cord to manikin
- Place manikin in delivery bed
- Make sure needed equipment is available on resuscitation table

Prepare screen

- Select Scenario Training from the menu
- Select participants from the learner list
- Select training level

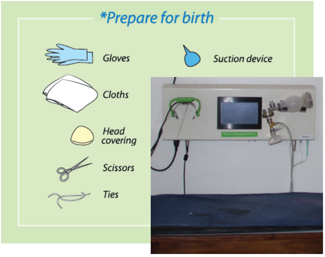

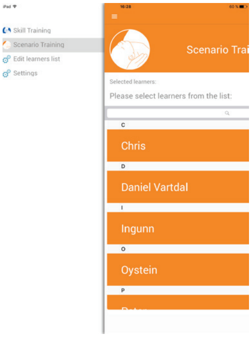

2 - Introduction

Brief participants on how to do scenario training

- We want to do this simulation as realistic as possible - work together as we do with real babies
- Time aspect is important - do all tasks as we would with real babies

Introduce scenario to participants

- Baby is not breathing after delivery
- Follow HBB action plan
- Start ventilations within 1 minute
- Ventilate until baby is breathing
- Observe heart rate during ventilation

Discuss roles and resopnsibilities with participants

- Who will keep track of time?
- Who will be responsible for ventilating the baby?

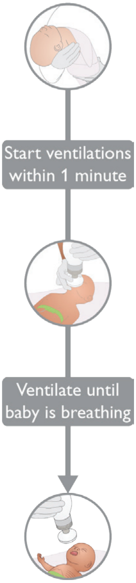

3 - Scenario

- Press "Baby is born" to start scenario
- Observe participants and log tasks on the screen

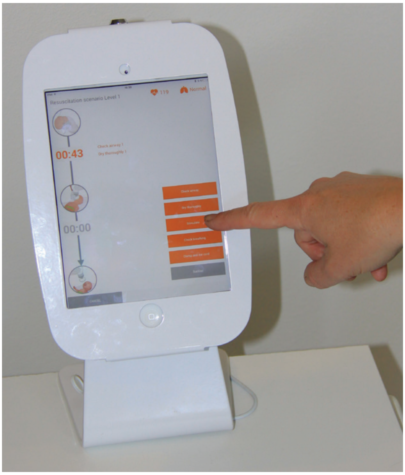

4 - Discussion

The discussion is an important part of the scenario training. Encourage participants to talk and share their experiences.

- What happened?
- What worked well?
- How did we work together?
- What can be improved?

A good advice is to repeat the scenario at least one more time after the discussion.

Master Trainer Guide **Rev 01**

Laerdal
helping save lives
