# Supplementary material for: Newborn resuscitation simulation training and changes in clinical performance and perinatal outcomes: a clinical observational study of 10,481 births
Source: Adv Simul (Lond). 2022 Nov 5;7:38. doi: 10.1186/s41077-022-00234-z (PMC9636744; doi:10.1186/s41077-022-00234-z)

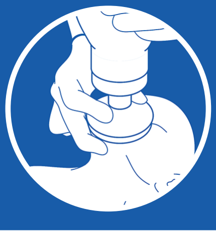

Ventilation Skill Training

How to use manikin and screen

1

Place the manikin on the resuscitation table and put on the green heart rate sensor

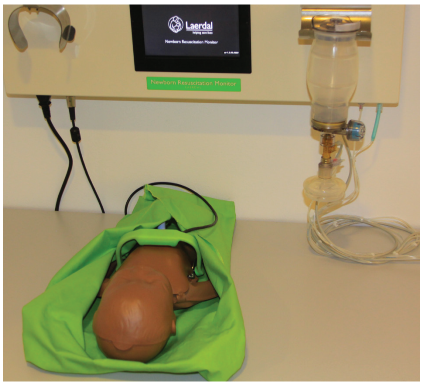

2

Activate the screen

Select your name from the list and the level you want to train

Read the introduction and start ventilations

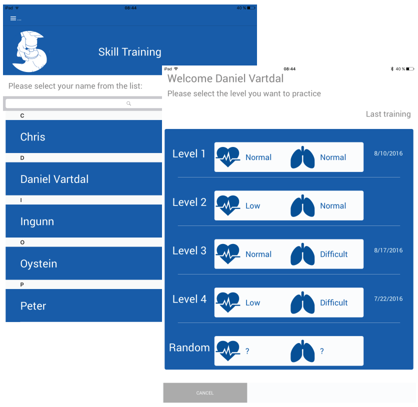

3

Start ventilation

Look for chest rise and observe heart rate on the heart rate monitor

Adjust ventilation technique if needed

The screen shows how long time you have been ventilating

Ventilate until the manikin starts crying

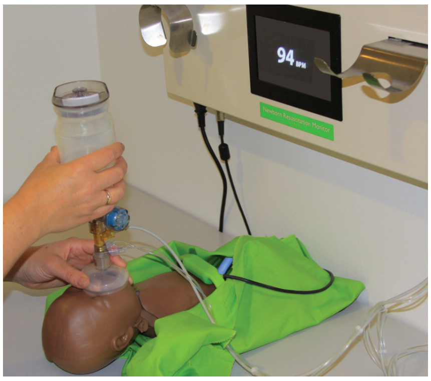

4

Recieve feedback on ventilation time and how to improve your ventilations

A good advice is to repeat the same level at least one more time

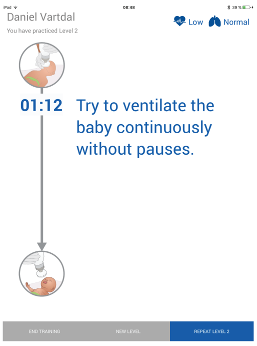

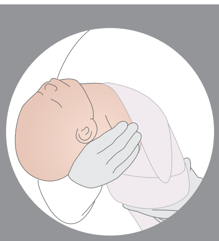

Newborn Resuscitation

How to ventilate newborn babies

Start ventilations within 1 minute after birth

Follow the HBB action plan

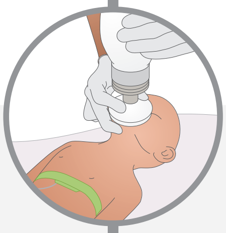

Ventilate until baby is breathing

Give 40-60 ventilations per minute
- Open airways
- Good mask seal

Look for chest rise
- Chest rise indicates that the baby gets air into the lungs
- Adjust ventilation technique if you don't see chest rise

Some babies need higher pressure initially for the lungs to open
Give a few ventilations with increased pressure

Observe heart rate
- **Low heart rate** indicates that the baby has had too little oxygen
- **Decreasing heart rate** indicates that the baby does not get enough air into the lungs
- **Increasing heart rate** indicates that the baby gets air into the lungs

Keep focus on ventilations
- The baby may need ventilations for a while to start breathing
- Avoid any unnecessary pauses

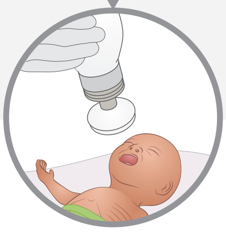

Supplement: Supplementary file 2 — Additional file 2. Learner Guide. A simple introduction folder for midwives about how to perform ventilation skill training. [file 41077_2022_234_MOESM2_ESM.pdf]
